# Enamel Caries in Young Adults and Progression From Adolescence: Fit Futures, a Longitudinal Cohort Study

**DOI:** 10.1016/j.identj.2026.109739

**Published:** 2026-07-15

**Authors:** Elin Hadler-Olsen, Aida Mulic, Anne-Sofie Furberg, Natalia Petrenya

**Affiliations:** a The Public Dental Health Service Competence Center of Northern Norway, Tromsø, Norway; b Department of Medical Biology, Faculty of Health Sciences, UiT the Arctic University of Norway, Tromsø, Norway; c Department of Clinical Dentistry, Faculty of Health Sciences, UiT the Arctic University of Norway, Tromsø, Norway; d Nordic Institute of Dental Material (NIOM), Oslo, Norway; e Department of Microbiology and Infection Control, University Hospital of North Norway, Tromsø, Norway; f Faculty of Health and Social Sciences, Molde University College, Molde, Norway

**Keywords:** Enamel caries, Progression, Remineralisation, Risk factors, Cohort study, Longitudinal, Adolescence, Young adulthood

## Abstract

**Introduction and aims:**

This study aimed to determine the extent and trajectory of enamel caries from adolescence through young adulthood and the associated factors.

**Methods:**

Data were from the first and third Fit Futures (FF) studies that followed a cohort of Norwegians from the first year of upper secondary school (FF1 2010-2011, age 17) for 10 years (FF3 2021-2022, age 27). Participants answered questionnaires and underwent clinical dental examinations. The study sample included participants with data on caries and restorations at both ages 17 and 27 (N = 584, 53% of the original FF1 cohort, 53% women). Data were analysed using descriptive statistics, and associations between risk factors and caries activity were examined using regression models.

**Results:**

Almost all participants had enamel caries lesions (median 12.0 and 9.0 lesions at ages 17 and 27, respectively), and 64% had lesions that had progressed into dentin during the observation period. At the tooth surface level, 10% of all surfaces had enamel caries at age 17, of which 18% had progressed to dentin caries by age 27. The highest progression rates were observed on proximal surfaces and for premolars of the upper jaw. In regression analyses, high caries activity was associated with female sex, low socioeconomic status, poorer self-reported general health, poor oral hygiene habits, and high soft drink intake.

**Conclusion and clinical relevance:**

Enamel caries lesions were almost ubiquitous, but more than 80% did not progress over a 10-year period, lending strong support to nonoperative treatment of such lesions. Nevertheless, the study demonstrated relatively high caries activity during the transition from adolescence to adulthood and underlines the importance of dietary guidance and oral hygiene motivation to prevent caries. Having medium or low socioeconomic status and facing general health issues should also be considered important caries risk factors.

## Introduction

The earliest visual manifestation of dental caries appears clinically as a chalky, white opacity, a so-called white spot lesion, or a radiolucency in the enamel on radiographs. Such initial caries lesions may progress deeper into dentin, leading to cavity formation and pulpal involvement. However, the balance between demineralisation and remineralisation can shift, allowing for arrest or remineralisation of carious lesions in the enamel, which forms the foundation of nonoperative treatment of enamel caries.^1^^,^^2^ This may involve hygiene instructions to more efficiently remove dental plaque, dietary guidance to restrict sugar intake, and various forms of fluoride treatment to reduce the enamel’s susceptibility to acid and promote remineralisation.

For epidemiologic purposes, caries is often reported using the World Health Organization’s Decayed (D), Missing (M), and Filled (F) index, where a tooth (T) or tooth surface (S) is considered decayed when caries reaches the dentin.^3^ However, setting the cutoff at dentin caries leads to underreporting of actual caries prevalence, caries risk, and treatment need. Additionally, excluding enamel caries from reports results in loss of data on the trajectory of enamel caries lesions over time and across life phases. Such information could advance our understanding and management of the disease. By including enamel caries lesions in epidemiologic studies, we can gain insights into the remineralisation, arrest, or progression of lesions, which are crucial for developing effective preventive strategies.

After tooth eruption, enamel matures and becomes less susceptible to demineralisation. Therefore, the dentition is inherently most prone to caries development during the first 2 to 4 years after eruption^4^ (eg, in childhood and adolescence). However, other factors influencing caries activity may change with age and life phases, such as oral hygiene habits—interest in cleaning the teeth and the ability to manage it efficiently, knowledge, dietary habits, general health and medication, salivary flow rate, and use of dental health services. These factors contribute to the caries activity over time.

The transition from adolescence to adulthood often involves significant life changes, such as moving out of the parental home, pursuing education, or starting a career.^5^ These changes may influence the financial situation, dietary habits, and the focus on oral health and, in turn, caries activity. In the present longitudinal study, the prevalence and trajectory of enamel caries were assessed in a cohort of young Norwegians from the age of 17, when the permanent teeth (third molars excepted) had erupted and undergone posteruptive maturation, until the age of 27. The aims were to determine enamel caries prevalence and extent at individual and tooth surface levels, track lesion trajectories from adolescence to young adulthood, and identify associated risk factors.

## Materials and methods

### Study design and population

This longitudinal study used data from the first and third waves of the Fit Futures (FF) cohort study (FF1 and FF3). In FF1 (2010-2011), all first-year upper secondary school (USS) students from Tromsø (urban) and Balsfjord (rural) municipalities in northern Norway (N = 1117) were invited (age about 17 years). The participants (N = 1038, 93%) completed questionnaires and underwent clinical health examinations, including a dental examination, as described in detail previously.^6^ In FF2 (2012-2013), all FF1 participants, along with any other third-year students at the same schools, were invited (age about 19 years). Dental examinations were not conducted in FF2. In FF3 (2021-2022), all participants from FF1 and/or FF2 (N = 1154) were invited and 715 (62.0%) participated (age about 27 years). FF3 included questionnaires and dental examinations similar to FF1.^7^ In the present study, participants with caries data recorded in both FF1 and FF3 (N = 591) were included, but 7 participants older than 31 years were excluded to have a more homogeneous cohort, leaving 584 (56.3% of the original FF1 cohort) for analysis. The mean (standard deviation (SD)) age of the participants was 16.6 (0.8) years in FF1 (denoted age 17) and 26.8 (0.8) years in FF3 (denoted age 27). Significantly more men, vocational USS program students, and participants with higher dentin caries prevalence dropped out compared to those who participated in both waves.^7^

This study was performed in line with the principles of the Declaration of Helsinki. Approval was granted by the Regional Committee for Health Research Ethics (REK 742265). Written informed consent was obtained from all individual participants included in the study.

### Clinical oral examinations

Clinical oral examinations included 4 bitewing (BW) radiographs and 9 intraoral photographs (Canon EOS 60D with Sigma EM-140 DG Macro Flash Canon Blitz). One dentist and 1 assistant performed most examinations (>90%), with backup teams handling the remainder. The examination teams differed between FF1 and FF3. All examiners underwent training and calibration prior to data collection.

### Caries registration

Caries registration procedures have been described previously for both FF1^8^ and FF3.^7^ Caries lesions were assessed from BWs and clinical photographs under standardised viewing conditions in a dark room. A 5-point grading scale was used for radiographic caries assessment on BWs: (1) caries in the outer half of the enamel, (2) caries in the inner half of the enamel, (3) caries in the outer third of the dentin, (4) caries in the middle third of the dentin, and (5) caries in the inner third of the dentin.^9^^,^^10^ Clinical photographs and a picture illustration guide^11^ supported the evaluation of occlusal, buccal, and lingual caries. For analysis, surfaces with caries grades 1 and 2 were classified as having enamel caries (D_E_S), and surfaces with caries grades 3 to 5 were classified as having dentin caries (D_D_S). Third molars were excluded from all analyses. One calibrated dentist recorded caries on all participants in FF1, and another calibrated dentist recorded caries on all participants in FF3.

### Registration of tooth restorations

Tooth restorations were recorded by the same 2 dentists who performed the caries registration in FF1 and FF3, based on BWs and photographs. Surfaces with permanent restorations were classified as filled (FS), whereas surfaces with temporary restorations were classified as having D_D_S. Fissure sealants were not recorded.

### Calibration and reliability

Calibration procedures for caries registration in FF1 and FF3 have been described previously.^7^^,^^8^ In brief, the principal investigators were calibrated with other experienced dentists prior to both studies. In FF1, the interobserver agreement for caries registration on BWs achieved a weighted κ of 0.7 (substantial agreement), while in FF3, the interobserver agreement yielded an intraclass correlation of 0.88 (good reliability). Intraobserver agreement for the principal investigator (dentist) for registrations based on information from both BWs and photographs was an intraclass correlation of 0.84 for caries registration and Cohen’s κ of 0.86 for restorations, based on the reevaluation of BWs and photographs from 10 participants after at least 2 months.

### Variables derived from caries and restoration data

**Figure 1:**
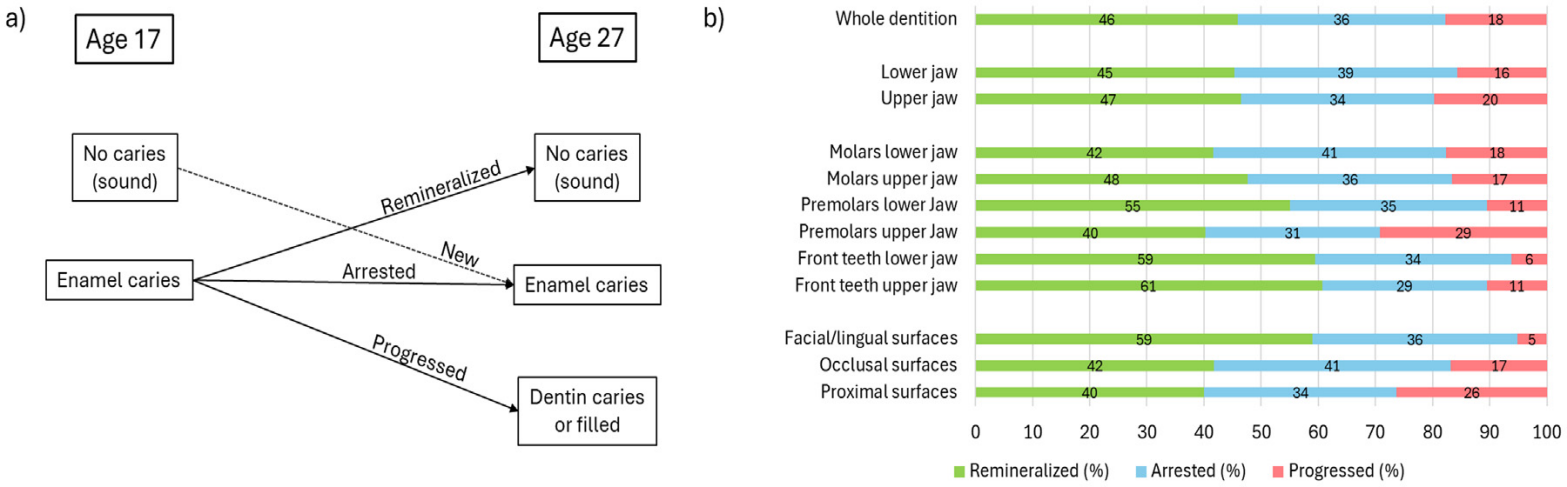
Trajectories of 7598 enamel caries lesions from ages 17 to 27. The Fit Futures cohort, N = 584. (a) The various trajectories of enamel caries lesions from ages 17 to 27. (b) Percentage of enamel caries lesions recorded at age 17 that were remineralised (in green), arrested (in blue), or progressed (in red) by age 27. Data are presented separately for the whole dentition, the lower and upper jaw, tooth groups of the upper and lower jaw, and various tooth surfaces.

The number and proportion of D_E_S were calculated at ages 17 (FF1) and 27 (FF3) at both participant and tooth surface levels. Surfaces that were recorded with enamel caries at age 17 and with dentin caries or a restoration at age 27 were classified as progressed (D_E_S progressed). Surfaces with enamel caries at both ages 17 and 27 were classified as arrested (D_E_S arrested), while those recorded with enamel caries at age 17 but sound at age 27 were classified as remineralised (D_E_S remineralised), as illustrated in Figure 1a. The proportion of surfaces with the various trajectories was calculated (number of remineralised/arrested/progressed lesions at age 27 divided by number of surfaces with enamel caries lesions at age 17 multiplied by 100). The number of surfaces with new enamel caries between ages 17 and 27 (D_E_S new) was determined by subtracting the number of arrested enamel caries surfaces (D_E_S arrested) from the total number of surfaces with enamel caries at age 27 (D_E_S 27 years). Additionally, the number of surfaces with D_D_S, permanent restorations (FS), and the combined total of surfaces with dentin caries or restorations (D_D_FS) were calculated at both ages.

### Handling of missing teeth

The reasons for missing teeth were not recorded. At age 27, a total of 273 teeth were missing, of which 231 (84.6%) were premolars, canines, or incisors. These are teeth that, in a young population with free access to dental services throughout childhood and adolescence, are usually missing for reasons other than caries, such as agenesis,^12^ orthodontic extractions,^13^ or dental trauma.^14^ Some of the missing molars had also been extracted for reasons other than caries, such as severe mineralisation defects.^15^ To avoid overestimating caries, we therefore excluded all teeth missing at age 27 from analyses.

### Questionnaire variables

The following covariates were derived from the questionnaires at age 17 (FF1) and/or age 27 (FF3):

**Sex** was recorded as either male or female.

**USS program** at age 17 was assessed with the following options: (1) academic, (2) sports, and (3) vocational. For some analyses, the academic and sports programs were combined.

**The highest level of education completed** at age 27 was assessed with the following options: (1) primary school, (2) completed vocational USS, (3) completed academic USS (including sports program), (4) college/university less than 4 years, and (5) college/university 4 years or more.

**The financial situation** at age 27 was assessed with the following options: (1) very difficult, (2) difficult, (3) average, (4) good, and (5) excellent. These were recategorised into 3 categories: difficult (original options 1 and 2), average (original option 3), and good (original options 4 and 5).

**Self-reported general health** at ages 17 and 27 was assessed using a 5-point Likert scale from (1) very poor to (5) very good. The variable was recategorised into 3 categories: poor (original options 1 and 2), moderate (original option 3), and good (original options 4 and 5). For regression analyses, the moderate and poor categories were combined to ensure sufficient group size.

**Toothbrushing frequency** at age 17 was assessed with the following options: (1) less than once a week, (2) once a week, (3) 2 to 3 times a week, (4) 4 to 6 times a week, (5) once a day, and (6) twice a day or more often. At age 27, the options were (1) less than once a week, (2) a few times a week, (3) once a day, and (4) twice a day or more often. To harmonise the data, both variables were recoded into 3 categories: less than once a day (original categories 1-4 at age 17, 1-2 at age 27), once a day, and twice a day or more often. For regression analyses, the variable was dichotomised into less than twice a day and at least twice a day.

Whether the participants had **regular dental visits** at age 27 was assessed with the following options: (1) yes, more than once a year; (2) yes, once a year; (3) yes, every second year; (4) yes, less than every second year; (5) no, only for acute problems; and (6) no, I never visit. These responses were recoded into 3 categories, which we used for regression analysis: at least every second year (options 1-3), less than every second year (option 4), and acute only or never (options 5 and 6). At age 17, all participants were offered regular, free recalls in the public dental health service.

Six cariogenic dietary variables were available for the present analysis. The detailed description is presented in Supplementary Table S1. The frequency of **intake of sugar-containing soft drinks** (later referred to simply as "soft drinks") at age 17 was assessed with the following options: (1) rarely/never, (2) 1 to 6 glasses per week, (3) 1 glass per day, (4) 2 to 3 glasses per day, (5) ≥4 glasses per day. Those at age 27 had the following options: (1) rarely/never, (2) 1 to 3 glasses per month, (3) 1 to 2 glasses per week, (4) 3 to 4 glasses per week, (5) 5 to 6 glasses per week, (6) 1 glass per day, (7) 2 to 3 glasses per day, and (8) ≥4 glasses per day. To align the coding, the options at age 27 were recoded into 5 categories: rarely/never (original options 1 and 2), 1 to 6 glasses per week (original options 3-5), 1 glass per day (original option 6), 2 to 3 glasses per day (original option 7), and ≥4 glasses per day (original option 8).

For regression analyses, a variable called socioeconomic status (SES) was computed based on the variables USS program, education, and financial situation, where having a combination of academic USS program, higher education of at least 4 years, and reporting a good financial situation was defined as high SES, and all other combinations were defined as medium/low SES.

### Statistical analysis

Statistical analyses were conducted using SPSS version 26 (IBM Corporation) and STATA version 18 (StataCorp LLC). Descriptive statistics summarised the data, including frequencies and prevalences for categorical variables, as well as medians with 25th (Q1) and 75th (Q3) percentiles and means with SDs for continuous variables. Nonparametric tests were applied to assess group differences due to the nonnormal distribution of the data.

### Outcome: number of enamel caries lesions (ages 17 and 27)

We converted the data from a wide format to a long format to analyse the factors associated with changes in the number of enamel caries lesions using mixed-effects models. Due to overdispersion, the negative binomial (NB) regression model was more suitable than the Poisson model. Consequently, univariable and multivariable generalised linear mixed-effects models with a negative binomial distribution were implemented using the STATA command menbreg. The model accounted for the hierarchical structure of the data (individuals nested within FF1 and FF3) by including a random intercept for each subject (ID) to address within-subject correlation from repeated measures. Robust standard errors ensured reliable inference. Time-invariant factors (sex, SES, and dental visits) and time-varying factors measured at ages 17 and 27 (general health, toothbrushing, soft drink intake, and D_D_FS) were included in the final model. The sensitivity analysis assessing the robustness of the model in relation to the other 5 cariogenic dietary variables available for this project, as well as the justification for using the soft drink intake variable in the final model, can be found in Supplementary Appendix 1, Tables S2 and S3. In the sensitivity analysis, all dietary variables were treated as categorical time-varying variables. Soft drink intake was a primary factor of interest also due to its theoretical basis and established dose-response relationship for influencing caries progression.^16^, ^17^, ^18^ However, longitudinal data on this relationship, particularly concerning the progression of initial caries, remain scarce.^19^ In our dataset, soft drink intake varied sufficiently within individuals over time, enabling the estimation of transition effects using mixed-effects regression models. In the final mixed-effects model, between-individual effects were calculated as the average soft drink intake across time points (ages 17 and 27), while within-individual effects were calculated as the deviation from this average at each time point.^20^ To account for nonlinear effects of D_D_FS on enamel caries lesions, spline terms with a knot at D_D_FS = 11 (75th percentile) were included, as this threshold distinguishes individuals with high caries experience. This approach allowed for different slopes below and above the knot. Residual diagnostics confirmed no violations of model assumptions, and multicollinearity was not detected. The random intercept variance indicated significant variability in enamel lesion numbers between individuals, justifying the use of a mixed-effects model. No significant interaction terms between time and risk factors were observed, indicating consistent effects at ages 17 and 27. The reduction in sample size due to missing values was minimal (5%) and primarily attributed to missing data in the dental visits variable. A sensitivity analysis excluding the dental visits variable is presented in Supplementary Appendix 2, Table S4 and yielded consistent results. Results are reported as incidence rate ratios (IRRs) with 95% confidence intervals (CIs) and *P* values.

### Outcome: number of progressed enamel caries lesions

Three models were used to analyse factors associated with progressed enamel caries lesions: logistic regression for the binary outcome (presence/absence of progression); NB regression for the count of progressed lesions, accounting for overdispersion; and zero-inflated negative binomial (ZINB) regression, modelling both the number of progression (count part) and the likelihood of no progression (zero-inflation part) to address excess zeros (36%). These 3 models were applied separately to baseline characteristics at age 17 (sex, USS program, general health, toothbrushing, and soft drink intake) and data at age 27 (sex, education, general health, toothbrushing, dental visits, and soft drink intake). The rationale for analysing associations between enamel caries progression between ages 17 and 27 and risk factors reported at the end of this period is that we do not know when potential changes in these risk factors have occurred between the 2 assessment points. Results were reported as odds ratios (ORs) with 95% CIs for logistic regression, IRRs with 95% CIs for NB regression, and IRRs (count part) and ORs (zero-inflation part) with 95% CIs for ZINB regression. Model fit was assessed using log-likelihood, Akaike information criterion, and Bayesian information criterion. The Vuong test compared ZINB and NB models to evaluate the impact of zero inflation. Justification of using the ZINB model and the choice of variables for the count and inflation parts are provided in Supplementary Appendix 3. Statistical significance was set at *P* < .05. All tests were 2-tailed.

## Results

### Enamel caries: prevalence and trajectory from adolescence to young adulthood, person level

Table 1 summarises the prevalence of caries at ages 17 and 27 at the person level and the trajectory of these lesions over time in a cohort of 584 Norwegians (52.7% women).

**Table 1:** Caries and restorations at the person level for ages 17 and 27 and enamel caries trajectories: the Fit Futures cohort, N = 584.

Characteristic	Prevalence (individuals with >0 affected surface), No. (%)	Number of affected surfaces, range	Number of affected surfaces, mean (SD)	Number of affected surfaces, median (Q1, Q3)	*P* value*
D_E_S					
Age 17	583 (99.8)	0-45	13.0 (7.7)	12.0 (7.0, 18.0)	<.001
Age 27	558 (95.5)	0-44	10.5 (7.6)	9.0 (5.0, 14.8)	
D_E_S progressed					
Age 17-27	376 (64.4)	0-18	2.3 (2.9)	1.0 (0.0, 3.0)	
D_E_S remineralised					
Age 17-27	560 (95.9)	0-26	5.9 (4.2)	5.0 (3.0, 8.0)	
D_E_S arrested					
Age 17-27	502 (86.0)	0-28	4.8 (4.2)	4.0 (1.0, 7.0)	
D_E_S new					
Age 17-27	520 (89.0)	0-23	5.7 (4.0)	4.0 (2.0, 9.0)	
D_D_S					
Age 17	218 (37.3)	0-16	0.9 (1.8)	0.0 (0.0, 1.0)	<.001
Age 27	295 (50.5)	0-18	1.6 (2.6)	1.0 (0.0, 2.0)	
FS					
Age 17	470 (80.5)	0-45	5.0 (5.5)	4.0 (1.0, 7.0)	<.001
Age 27	508 (87.0)	0-47	8.3 (8.1)	6.0 (2.0, 11.0)	
D_D_FS					
Age 17	482 (82.5)	0-47	5.8 (6.3)	4.0 (1.0, 8.0)	<.001
Age 27	517 (88.5)	0-53	9.9 (9.3)	8.0 (3.0, 14.0)	

Abbreviations: D_D_, dentin caries; D_D_FS, sum of surfaces with dentin caries or restorations; D_E_, enamel caries; D_E_S arrested, surfaces with enamel caries at both ages 17 and 27; D_E_S new, surfaces that developed enamel caries between ages 17 and 27; D_E_S progressed, surfaces with enamel caries at age 17 recorded with dentin caries or restorations at age 27; D_E_S remineralised, surfaces with enamel caries at age 17 recoded sound at age 27; FS, filled surfaces; Q1, quartile 1; Q3, quartile 3; S, surface.

⁎ Related-samples Wilcoxon signed rank test.

At age 17, all but 1 participant (99.8%) had at least 1 surface with enamel caries, with a median of 12.0 surfaces. By age 27, all but 26 participants (99.5%) had enamel caries, with a significantly lower median of 9.0 surfaces (*P* < .001). Over the same period, the median number of surfaces with dentin caries or restorations increased, as previously reported.^7^ The median number of new enamel caries lesions at age 27 was 4.0. Sixty-four percent of the participants had at least 1 enamel caries lesion at age 17 that had progressed into dentin or had a restoration by age 27. The maximum number of progressed lesions was 18, with a median of 1.0. From ages 17 to 27, 95.9% of individuals had remineralised enamel caries lesions, 86.0% had arrested lesions, and 89.0% of individuals had new lesions developed (Table 1).

### Enamel caries: prevalence and trajectory from adolescence to young adulthood—jaw, tooth group, and tooth surface level

Table 2 presents the prevalence of enamel caries at ages 17 and 27 on a surface level and the prevalence of new enamel caries lesions developed between ages 17 and 27.

**Table 2:** Enamel caries lesions at ages 17 and 27 by jaw, tooth groups, and tooth surfaces: the Fit Futures cohort, N = 584.

Surface	Enamel caries at age 17, No. (%)	Enamel caries at age 27, No. (%)	New enamel caries at age 27, No. (%)
Total (N = 73,422)	7598 (10.3)	6257 (8.5)	3498 (4.8)
Jaw			
Upper (n = 36,630)	3911 (10.7)	3283 (9.0)	1963 (5.4)
Lower (n = 36,792)	3687 (10.0)	2974 (8.1)	1535 (4.2)
Tooth groups			
Molars upper jaw (n = 11,590)	2544 (21.9)	1799 (15.5)	892 (7.7)
Molars lower jaw (n = 11,565)	2662 (23.0)	2057 (17.8)	972 (8.4)
Premolars upper jaw (n = 11,120)	1100 (9.9)	1037 (9.3)	701 (6.3)
Premolars lower jaw (n = 11,235)	993 (8.8)	836 (7.4)	493 (4.4)
Front upper jaw (n = 13,920)	267 (1.9)	447 (3.2)	370 (2.6)
Front lower jaw (n = 13,992)	32 (0.2)	81 (0.6)	70 (0.5)
Tooth surfaces			
Proximal (n = 32,160)	3416 (10.6)	2496 (7.8)	1346 (4.2)
Occlusal (n = 9102)	2000 (22.0)	1953 (21.5)	1125 (12.4)
Facial/lingual (n = 32,160)	2182 (6.8)	1817 (5.6)	1036 (3.2)

Enamel caries at age 17 and 27: the total number of surfaces with enamel caries lesions at that age. New enamel caries at age 27: number of surfaces that developed enamel caries between ages 17 and 27.

At age 17, 10.3% of all tooth surfaces had enamel caries. At age 27, this was reduced to 8.5% of all surfaces, of which 55.9% of the enamel lesions had developed between ages 17 and 27 (new enamel caries lesions). The distribution of enamel caries lesions between the different groups of teeth and tooth surfaces was similar at ages 17 and 27. Of the tooth groups, enamel caries was most prevalent in molars, particularly in the lower jaw, and least prevalent in frontal teeth (incisors and canines), with the lowest prevalence in the lower jaw. Of tooth surfaces, the occlusal surface had the highest prevalence of enamel caries, while the facial and lingual surfaces had the lowest. The number of enamel caries lesions decreased across all tooth surfaces and tooth groups by age 27, except for the front segments, where a slight increase was observed. Molars had the highest proportion of new enamel caries lesions, while teeth in the front segments had the lowest. Among tooth surfaces, the occlusal surface had the highest proportion of new enamel caries lesions (12.4%, Table 2).

Figure 1a and 1b illustrates different trajectories of enamel caries lesions from ages 17 to 27 and shows the proportion of enamel caries lesions with the various trajectories during this period by jaw, tooth groups, and tooth surfaces.

By age 27, 17.7% of enamel caries lesions recorded at age 17 had progressed to dentin caries or restorations, 36.3% remained unchanged (arrested), and 45.9% were remineralised. Progression was more common in the upper jaw, while frontal teeth in the lower jaw showed the least progression. Remineralisation was most frequent in the frontal teeth and on the facial or lingual surfaces. Of the various surfaces, the proximal surface had the highest progression rate, whereas the facial and lingual surfaces had the lowest progression rate (Figure 1b).

### Enamel caries and its trajectory from adolescence to young adulthood by participant characteristics

#### Descriptive analyses

The median number of enamel caries lesions at ages 17 and 27, as well as the number of progressed lesions during this period, varied according to participant characteristics (Table 3).

**Table 3:** Enamel caries and its trajectory from ages 17 to 27 by participant characteristics: the Fit Futures cohort, N = 584.

Characteristic	All	D_E_ age 17, median (Q1, Q3)	*P* value	D_E_ age 27, median (Q1, Q3)	*P* value	D_E_ progressed, median (Q1, Q3)	*P* value
Sex							
Female	308 (52.7)	12.0 (7.0, 18.0)	.931	9.0 (5.0, 14.0)	.959	1.0 (0.0, 3.0)	.845
Male	276 (47.3)	12.0 (7.0, 18.0)	Ref	9.0 (4.0, 15.0)	Ref	1.0 (0.0, 3.0)	Ref
USS program age 17							
USS academic	273 (46.7)	10.0 (6.0, 15.0)	Ref	8.0 (4.0, 13.0)	Ref	1.0 (0.0, 3.0)	Ref
USS sports	62 (10.6)	10.0 (6.0, 14.0)	1.000	7.5 (2.8, 12.0)	.801	1.0 (0.0, 3.0)	1.000
USS vocational	249 (42.6)	**15.0 (9.0, 21.0)**	**<.001**	**11.0 (6.0, 18.0)**	**<.001**	**2.0 (0.0, 5.0)**	**<.001**
Education age 27							
Higher education long	156 (28.1)			7.0 (3.0, 11.0)	Ref	1.0 (0.0, 2.0)	Ref
Higher education short	151 (27.2)			9.0 (4.0, 15.0)	.247	**1.0 (0.0, 3.0)**	**.032**
USS academic/sport	122 (22.0)			**10.5 (6.0, 16.0)**	**.004**	**2.0 (0.0, 4.0)**	**.001**
USS vocational	92 (16.6)			**9.0 (5.0, 16.0)**	**.035**	**2.0 (0.0, 4.0)**	**.001**
Secondary school	34 (6.1)			**11.5 (9.0, 20.0)**	**<.001**	**3.0 (1.0, 5.0)**	**<.001**
Finances age 27							
Good	284 (51.2)			8.0 (3.0, 12.0)	Ref	1.0 (0.0, 3.0)	Ref
Average	196 (35.3)			**10.0 (6.0, 16.0)**	**<.001**	**2.0 (0.0, 4.0)**	**.008**
Difficult	75 (13.5)			**11.0 (6.0, 18.0)**	**.001**	**2.0 (0.0, 4.0)**	**.025**
General health age 17							
Good	427 (74.0)	11.0 (7.0, 17.0)	Ref	8.0 (4.0, 13.0)	Ref	1.0 (0.0, 3.0)	Ref
Average	115 (19.9)	**14.0 (8.0, 20.0)**	**.028**	**11.0 (6.0, 17.0)**	**.006**	**2.0 (0.0, 4.0)**	**.020**
Poor	35 (6.1)	**15.0 (10.0, 24.0)**	**.039**	**12.0 (6.0, 21.0)**	**.040**	**4.0 (1.0, 6.0)**	**.001**
General health age 27							
Good	411 (74.1)			9.0 (4.0, 13.0)	Ref	1.0 (0.0, 3.0)	Ref
Average	111 (20.0)			**12.0 (8.0, 18.0)**	**<.001**	**2.0 (0.0, 4.0)**	**.020**
Poor	33 (5.9)			5.0 (2.0, 14.0)	.664	1.0 (0.0, 4.5)	1.000
Toothbrushing age 17							
≥2/day	378 (65.4)	11.0 (6.0, 16.0)	Ref	8.0 (4.0, 13.0)	Ref	1.0 (0.0, 3.0)	Ref
1/day	134 (23.2)	11.0 (7.8, 20.0)	.232	9.0 (5.0, 15.0)	.486	**2.0 (0.8, 4.0)**	**<.001**
<1/day	66 (11.4)	**19.0 (12.3, 26.0)**	**<.001**	**14.5 (8.0, 20.8)**	**<.001**	**4.0 (2.0, 6.0)**	**<.001**
Toothbrushing age 27							
≥2/day	402 (72.4)			8.0 (4.0, 13.0)	Ref	1.0 (0.0, 3.0)	Ref
1/day	127 (22.9)			**11.0 (6.0, 16.0)**	**.016**	**2.0 (1.0, 5.0)**	**<.001**
<1/day	26 (4.7)			12.0 (6.8, 18.5)	.056	**3.0 (1.0, 5.0)**	**.003**
Dental visits age 27							
≥ every second year	313 (56.4)			9.0 (4.0, 13.0)	Ref	1.0 (0.0, 3.0)	Ref
< every second year	104 (18.7)			8.0 (4.0, 14.0)	1.000	1.0 (0.0, 3.0)	1.000
Never/acute only	138 (24.9)			**11.0 (6.0, 17.0)**	**.009**	2.0 (0.0, 4.0)	.933
Soft drinks age 17							
Rarely/never	136 (23.6)	11.0 (7.0, 16.0)	Ref	9.0 (5.0, 13.0)	Ref	1.0 (0.0, 3.0)	Ref
1-6 glasses/week	320 (55.6)	11.0 (7.0, 18.0)	1.000	8.0 (4.0, 14.0)	1.000	1.0 (0.0, 3.0)	.870
1 glass/day	59 (10.2)	15.0 (8.0, 20.0)	.468	11.0 (7.0, 17.5)	.173	2.0 (0.0, 4.0)	.193
≥2 glasses/day	61 (10.6)	13.0 (9.0, 21.5)	.114	11.0 (5.5, 18.5)	.392	2.0 (0.5, 4.0)	.058
Soft drinks age 27							
Rarely/never	403 (73.5)			9.0 (4.0, 13.0)	Ref	1.0 (0.0, 3.0)	Ref
1-6 glasses/week	125 (22.8)			10.0 (5.0, 15.5)	.397	2.0 (0.0, 4.0)	.108
1 glass/day	12 (2.2)			7.5 (3.3, 16.0)	1.000	1.5 (0.0, 3.8)	1.000
≥2 glasses/day	8 (1.3)			18.0 (9.5, 21.0)	.098	4.0 (1.0, 5.0)	.351

Abbreviations: D_E_, enamel caries; D_E_ progressed, surfaces with enamel caries at age 17 that were recorded with dentin caries or a restoration at age 27; USS, upper secondary school.

Differences between variable categories were assessed with independent-samples Mann-Whitney *U* tests for variables with 2 categories and with independent-samples Kruskal-Wallis tests for variables with more than 2 categories, with Bonferroni correction for multiple comparisons. The reference group is indicated with “Ref”. Numbers in bold indicate statistically significant associations.

The median number of enamel caries lesions did not differ by sex at either age 17 or 27. Participants enrolled in academic USS programs (vs. vocational) and those who had completed 4 years or more of higher education exhibited relatively low numbers of enamel caries lesions and progressed lesions. Similarly, participants reporting a good financial situation at age 27 had lower median numbers of enamel caries lesions, as well as fewer progressed lesions, compared to those with an average or difficult financial situation (Table 3).

Participants reporting moderate or poor health at age 17 or 27 also had relatively high numbers of enamel caries lesions, as well as a higher number of progressed lesions between ages 17 and 27, than those with good general health. Conversely, higher toothbrushing frequency was associated with fewer enamel caries lesions and progressed lesions. Reporting dental visits for acute problems only or never at age 27 was associated with high numbers of enamel caries lesions at age 27 (Table 3).

#### Regression analysis

##### Outcome: number of enamel caries lesions at ages 17 and 27

Risk factors associated with the number of enamel caries lesions over time, assessed using univariable (unadjusted) and multivariable mixed-effects NB regression models, are presented in Table 4.

**Table 4:** Mixed-effects models for number of enamel caries lesions, ages 17 to 27.

Number of enamel caries lesions (ages 17 and 27)	Univariable model		Multivariable model, n = 555	
	IRR (95% CI)	*P* value	IRR (95% CI)	*P* value
Sex				
Female	Reference		Reference	
Male	1.00 (0.90-1.10)	.921	**0.89 (0.80-0.98)**	**.023**
SES*				
High	Reference		Reference	
Medium/low	**1.31 (1.17-1.47)**	**<.001**	**1.12 (1.01-1.26)**	**.040**
General health				
Good	Reference		Reference	
Moderate/poor	**1.15 (1.05-1.25)**	**.002**	**1.10 (1.01-1.19)**	**.036**
Toothbrushing				
≥2/day	Reference		Reference	
<2/day	**1.25 (1.14-1.36)**	**<.001**	**1.13 (1.03-1.23)**	**.007**
Dental visits				
≥1/2 year	Reference		Reference	
<1/2 year	1.02 (0.89-1.17)	.786	1.03 (0.92-1.16)	.619
Acute/never	**1.19 (1.05-1.34)**	**.005**	**1.15 (1.03-1.28)**	**.013**
Soft drinks (between-effect)^†^				
Rarely/never	Reference		Reference	
1-6 glasses/week	1.02 (0.89-1.18)	.734	1.08 (0.93-1.24)	.332
1 glass/day	1.29 (0.96-1.74)	.097	**1.35 (1.02-1.80)**	**.039**
≥2 glasses/day	**1.56 (1.22-2.00)**	**<.001**	**1.37 (1.04-1.80)**	**.025**
Soft drinks (within-effect)†				
Rarely/never	Reference		Reference	
1-6 glasses/week	**1.19 (1.09-1.29)**	**<.001**	**1.32 (1.20-1.45)**	**<.001**
1 glass/day	1.09 (0.94-1.27)	.245	**1.37 (1.16-1.62)**	**<.001**
≥2 glasses/day	**1.21 (1.03-1.42)**	**.017**	**1.46 (1.22-1.74)**	**<.001**
D_D_FS‡ spline (knot at D_D_FS = 11)				
D_D_FS (spline 1)	**1.04 (1.02-1.05)**	**<.001**	**1.04 (1.02-1.05)**	**<.001**
D_D_FS (spline 2)	**1.01 (1.01-1.02)**	**<.001**	1.01 (1.00-1.01)	.064

Abbreviations: CI, confidence interval; D_D_FS, dentin caries decayed and filled surfaces; IRR, incidence rate ratio; SES, socioeconomic status.

The absolute number of participants for each univariable model is as follows: sex (n = 584), socioeconomic status (n = 584), general health (n = 582), toothbrushing (n = 582), dental visits (n = 555), soft drink intake (n = 582), and D_D_FS (n = 584). Missing values were treated by listwise deletion in the multivariable model (5% reduction in sample size). Numbers in bold indicate statistically significant associations.

⁎ SES was computed based on the variables upper secondary school program, education, and financial situation, where having a combination of an academic upper secondary school program, higher education of at least 4 years, and reporting a good financial situation was defined as high SES, and all other combinations were defined as medium/low SES.

† Between-individual effect: modelled by taking the average soft drink intake for each person across all time points. Within-individual effect: modelled by calculating the deviation of each observation from that individual’s average at each time point.

‡ D_D_FS index estimates sum of decayed-filled surfaces or permanent restorations.

In the multivariable model, we found that being male reduced the number of enamel caries lesions by 11% compared to females, while no association was detected in the univariable model. Lower SES and moderate or poor general health increased the number of enamel caries lesions by 12% and 10%, respectively. Visiting the dentist only for acute issues or never increased the number of enamel caries lesions by 15% compared to regular visits. Those who brushed their teeth less than twice a day had a 13% higher number of enamel caries lesions than those who brushed at least twice a day. Individuals who on average consumed 1 or ≥2 glasses of soft drinks per day had a 35% and 37% higher number of enamel caries lesions, respectively, compared to those who consumed <1 glass per week (Table 4).

##### Within-subject effect for soft drink intake

The within-individual effect captures how changes in soft drink intake over time (deviation from an individual’s average intake) are associated with the number of enamel caries lesions.

In multivariable models, when individuals increased their soft drink intake to 1-6 glasses per week, the number of enamel caries lesions increased by 32% relative to their own average consumption. Similarly, when individuals increased their consumption to 1 or ≥2 glasses per day, the number of enamel caries lesions increased by 37% and 46%, respectively (Table 4).

### Sensitivity analyses of soft drink intake associations in relation to other dietary variables

Sensitivity analyses with the detailed analysis of 6 dietary determinants of caries are reported in Supplementary Appendix 1 Tables S2 and S3 (models 1-7). In the univariable models (Supplementary Table S2), soft drinks, fruit juice, fruit drinks (sweetened), and snacks showed significant positive associations with the number of enamel caries lesions, while fruits and sweets did not. However, when all dietary variables were introduced simultaneously in the multivariable (mutually adjusted) model (Supplementary Table S2), only soft drinks remained consistently significant and exhibited a dose-response relationship.

Soft drinks, fruit juice, fruit drinks, and snacks also remained significant in models that included all other risk factors (ie, sex, SES, general health, toothbrushing, dental visits, and D_D_FS) when introduced separately (Supplementary Table S3, models 1, 2, 3, and 6). According to model 1 in Supplementary Table S3, relative to those who consumed soft drinks rarely/never (<1 glass per day), consuming 1-6 glasses per week, 1 glass per day, and ≥2 glasses per day was associated with 23%, 37%, and 44% higher number of enamel caries lesions, respectively. The association remained significant when the other 5 dietary determinants were included as well (Supplementary Table S3, model 7). Thus, soft drink intake showed the most consistent associations among dietary factors.

***Outcome: progressed enamel caries lesions***

### Baseline risk factors assessed at age 17 and progression of enamel caries lesions into dentin caries

Table 5 shows associations between risk factors assessed at age 17 and enamel lesion progression as a dichotomous or count variable estimated using multivariable logistic, NB, and ZINB regressions. Univariable logistic and NB regression models are shown in Supplementary Appendix 4, Table S5.

**Table 5:** Association between baseline risk factors assessed at age 17 and progression of enamel caries lesions into dentin caries, n = 571.

Characteristic	Multivariable models					
	Logistic		Negative binomial		Zero-inflated negative binomial	
	OR (95% CI)	*P* value	IRR (95% CI)	*P* value	IRR (95% CI)	*P* value
Sex						
Female	Reference		Reference		Reference	
Male	0.77 (0.52-1.15)	.201	**0.70 (0.56-0.89)**	**.003**	0.82 (0.66-1.01)	.059
General health						
Good	Reference		Reference		Reference	
Moderate/poor	1.41 (0.91-2.18)	.129	1.25 (0.99-1.57)	.058	**1.40 (1.13-1.72)**	**.002**
Soft drinks						
Rarely/never	Reference		Reference		Reference	
1-6 glasses/week	1.19 (0.77-1.86)	.433	1.13 (0.86-1.47)	.380	1.16 (0.89-1.51)	.259
≥1 glass/day	1.62 (0.88-2.98)	.120	1.30 (0.92-1.82)	.133	**1.44 (1.04-1.97)**	**.026**
					Zero-inflation part: likelihood of having no progression	
					OR (95% CI)	*P* value
USS program						
USS academic/sport	Reference		Reference		Reference	
USS vocational	**1.86 (1.26-2.73)**	**.002**	**1.57 (1.27-1.94)**	**<.001**	**0.34 (0.15-0.77)**	**.010**
Toothbrushing						
<2/day	Reference		Reference		Reference	
≥2/day	**0.41 (0.27-0.62)**	**<.001**	**0.54 (0.43-0.67)**	**<.001**	**7.12 (1.22-41.66)**	**.029**
Log-likelihood	–348.168		–1121.633		–1127.317	
AIC	710.34		2259.27		2272.63	
BIC	740.77		2294.05		2311.76	

Abbreviations: AIC, Akaike information criterion; BIC, Bayesian information criterion; CI, confidence interval; IRR, incidence rate ratio; OR, odds ratio; USS, upper secondary school.

The Vuong test of zero-inflated negative binomial versus negative binomial regressions, *P* = .001. Numbers in bold indicate statistically significant associations.

Brushing twice a day and participating in academic or sports USS programs significantly reduced the odds and counts of progressed enamel caries lesions. These factors also increased the likelihood of belonging to the “not-at-risk” or “always zero” group, with consistent results across all models. Moderate or poor general health was consistently associated with a higher number of progressed enamel caries lesions, becoming a significant factor in the ZINB model, where it was linked to a 40% increase in the number of progressed lesions (*P* = .002, Table 5). Additionally, consuming ≥1 glass of soft drinks per day was identified as a significant risk factor for enamel caries lesions progression in the ZINB model, with a 44% increase in the number of progressed enamel caries lesions (*P* = .026). Men showed fewer progressed enamel caries lesions in the NB model, although this result was less consistent across models (Table 5). A comparison of the NB and ZINB model fits, along with the interpretation of the model results for factors assessed at age 17, is provided in Supplementary Appendix 5.

### Association between risk factors assessed at age 27 and progression of enamel caries lesions into dentin caries

Table 6 shows the associations between risk factors assessed at age 27 and enamel caries lesion progression as a dichotomous or count variable estimated using multivariable logistic, NB, and ZINB regressions. Univariable logistic and NB regression models are shown in Supplementary Appendix 6, Table S6.

**Table 6:** Association between risk factors assessed at age 27 and enamel caries lesions progression, n = 548.

Characteristic	Multivariable models					
	Logistic		Negative binomial		Zero-inflated negative binomial	
	OR (95% CI)	*P* value	IRR (95% CI)	*P* value	IRR (95% CI)	*P* value
Sex						
Women	Reference		Reference		Reference	
Men	0.86 (0.58-1.27)	.439	0.80 (0.63-1.01)	.057	0.92 (0.75-1.13)	.425
General health						
Good	Reference		Reference		Reference	
Moderate/poor	1.07 (0.69-1.67)	.752	1.07 (0.83-1.39)	.579	**1.29 (1.03-1.61)**	**.027**
Soft drinks						
Rarely/never	Reference		Reference		Reference	
≥1/week	1.44 (0.92-2.26)	.113	1.13 (0.88-1.45)	.346	1.16 (0.92-1.45)	.214
Dental visits						
≥1/2 year	Reference		Reference		Reference	
<1/2 year	1.30 (0.48-1.24)	.285	0.76 (0.57-1.02)	.064	0.76 (0.58-1.00)	.054
Acute/never	1.09 (0.59-1.44)	.711	0.89 (0.69-1.15)	.382	0.92 (0.72-1.16)	.473
					Zero-inflation part: likelihood of having no progression	
					OR (95% CI)	*P* value
Education						
Higher ≥4 years	Reference		Reference		Reference	
Secondary school	**3.05 (1.14-8.17)**	**.027**	**2.03 (1.25-3.31)**	**.004**	0.17 (0.009-3.16)	.232
USS academic/sport	**2.12 (1.26-3.55)**	**.004**	**1.76 (1.29-2.40)**	**<.001**	**0.26 (0.08-0.83)**	**.022**
USS vocational	**2.09 (1.16-3.74)**	**.013**	**1.81 (1.28-2.55)**	**.001**	**0.29 (0.09-0.95)**	**.040**
Higher <4 years	**1.85 (1.15-2.95)**	**.011**	**1.48 (1.10-1.98)**	**.010**	**0.41 (0.19-0.91)**	**.026**
Toothbrushing						
<2/day	Reference		Reference		Reference	
≥2/day	**0.49 (0.31-0.78)**	**.003**	**0.64 (0.49-0.83)**	**.001**	4.22 (0.91-19.71)	.065
Log-likelihood	–357.912		–1101.527		–1083.993	
AIC	701.84		2182.13		2184.59	
BIC	749.21		2233.80		2240.58	

Abbreviations: AIC, Akaike information criterion; BIC, Bayesian information criterion; CI, confidence interval; IRR, incidence rate ratio; OR, odds ratio; USS, upper secondary school.

The Vuong test of zero-inflated negative binomial versus negative binomial regressions, *P* = .002. Numbers in bold indicate statistically significant associations.

Brushing twice a day and having a university education of ≥4 years significantly reduced the odds and number of progressed enamel caries lesions. Average or poor general health was associated with a higher number of progressed enamel caries lesions in the ZINB model, with a 29% increase in lesions (*P* = .027, Table 6). A comparison of the NB and ZINB model fits, along with the interpretation of the model results for factors assessed at age 27, is provided in Supplementary Appendix 7.

## Discussion

The present study showed that over a 10-year period, enamel caries was almost ubiquitous among adolescents and young adults. The number of enamel caries lesions that either progressed or remineralised between ages 17 and 27 was higher than the number of new lesions that developed, causing an overall reduction in enamel caries prevalence over time. On an individual level, 64% had enamel caries lesions that progressed to dentin caries or were restored over the study period; at the surface level, 18% of the lesions progressed. Also, 89% of the study participants developed new enamel caries lesions between ages 17 and 27, with a median of 4 lesions. Along with the increased prevalence of dentin caries and filled surfaces over the study period, this demonstrates considerable caries activity in this population. Nevertheless, most enamel caries lesions did not progress, which underscores the relevance of nonoperative treatment of initial caries and strongly supports holding off on operative treatment.

The relatively high caries activity of the cohort followed in the current study is in accordance with a recent study that analysed the global burden of untreated caries from 1990 to 2019 based on data from the Global Burden of Disease study.^21^ The study found that the incidence of caries in permanent teeth peaked in the 20- to 30-year age groups. We found a very high prevalence of enamel caries. Our results align well with those from a systematic review with meta-analyses that found a 90% prevalence of enamel caries in 16- to 19-year-olds.^22^ The somewhat higher prevalence found in the present study may be due to our choice to examine all tooth surfaces using a combination of radiographs and high-resolution clinical photographs, whereas many previous studies on enamel caries relied on radiographic evaluation of proximal surfaces only or on clinical examination alone.^22^^,^^23^

The most fundamental etiologic factors for caries are dental plaque and sugar consumption. Accordingly, we found that brushing teeth twice a day or more was associated with fewer enamel caries lesions over a 10-year period and a reduced progression of such lesions into dentin caries. Nevertheless, the effect appeared stronger at age 17 than at age 27, which may be due to improved oral hygiene habits recorded at age 27. Also, the association between enamel caries lesions and intake of soft drinks was robust. The mixed-effect model demonstrated a strong effect on the rate of enamel caries lesions for both long-term soft drink intake at the group level (between-subject variability) and for increases in soft drink intake for an individual (within-subject variability). A dose-response relationship was observed, with higher soft drink intake associated with a greater number of enamel caries lesions, after adjustment for other factors. Daily intake of soft drinks showed the strongest association with both an increased number of enamel caries lesions and their progression, accounting for approximately a 40% increase. For enamel lesion progression, soft drink intake at age 27 appeared to be a less significant factor compared to at age 17, likely due to reduced intake at older age. Our results are not surprising, but they highlight the importance of promoting good oral hygiene practice and dietary guidance to improve oral health into adulthood.

Although not statistically significant in the univariable model, women had a higher number of enamel caries lesions than men over a 10-year period in the mixed-effects model, which was controlled for other factors. A similar trend was observed across univariable models for enamel lesion progression, which showed no significant associations between sex and enamel lesion progression. However, in the multivariable NB model, females exhibited greater progression than males. This finding suggests that other factors, such as differences in oral hygiene practices, dietary habits, or access to dental care, act as negative confounders, influencing the observed sex differences. Many studies find that women are more caries active than men,^24^ with multifactorial, underlying reasons, including differences in salivary flow rate and composition, microbiome, hormonal factors and pregnancy, and socioeconomic factors.^25^ Although sex itself is not a modifiable factor, sex differences are important to acknowledge, and underlying modifiable factors should be addressed. Our study also confirmed other well-known risk factors for high caries activity, including medium or low SES and moderate or poor general health, highlighting the importance of more individualized and targeted efforts for these groups.

The study found that the D_D_FS index, which measures cumulative caries experience, was positively associated with an increase in enamel caries lesions over time. As a time-varying variable (measured at baseline and after 10 years), increasing D_D_FS scores were linked to a greater rise in enamel caries lesions from adolescence to young adulthood. However, this relationship was not linear. While higher D_D_FS scores were generally associated with more enamel caries lesions, individuals in the top 25% of D_D_FS scores showed a less pronounced increase. This may be due to a significant number of their teeth already being affected by decay or restorations, reducing the available enamel surface for new lesions. Furthermore, individuals with high D_D_FS scores may have undergone protective dental interventions and adopted better oral hygiene and dietary habits. Differences in enamel composition or anatomic structure, such as thinner enamel in these individuals, could also allow faster progression to dentin, bypassing visible enamel caries lesions. Unmeasured genetic factors could influence the observed findings in this study. Although the role of genetics in caries risk has been investigated in several studies,^26^, ^27^, ^28^, ^29^, ^30^ it remains unknown.

The prevalence and trajectory of enamel carious lesions varied across tooth surfaces. Proximal surfaces had the highest rate of progressed lesions. The high caries activity of these surfaces may be due to food retention between the surfaces, combined with their limited access for cleaning. The close contact between the proximal surfaces of adjacent teeth also makes them more prone to iatrogenic damage upon drilling of the neighbouring surface.^31^

Occlusal surfaces had the highest prevalence of enamel caries lesions at both ages 17 and 27 and the highest fraction of arrested and new enamel caries lesions. The occlusal surfaces have complex groove-fossa systems, where plaque can accumulate, especially during eruption, when access to and mechanical function of the surfaces are limited. After full eruption, the teeth are more accessible to cleaning and thereby less susceptible to caries,^32^ which may explain the high occurrence of arrested enamel caries on these surfaces. A previous study following caries incidence on proximal and occlusal surfaces from adolescence to young adulthood found that the incidence of dentin lesions on occlusal surfaces was higher in adolescence than in young adulthood, whereas the opposite was seen for proximal surfaces.^23^ This is in accordance with our findings that progression into dentin caries was most common on proximal surfaces.

Remineralisation, defined as surfaces that were recorded with enamel caries at age 17 but without caries or restorations at age 27, was most often seen on buccal and lingual surfaces. Some of these surfaces are situated close to salivary excretory ducts and benefit from the caries-protective effects of saliva. However, these surfaces are also easily accessible for brushing and other mechanical forces, as well as erosive substances. We did not measure mineral gain, and a substantial proportion of the lesions that were classified as remineralised had probably lost the porous demineralised layer due to mechanical or erosive tooth wear rather than actual mineral gain. Thus, our use of the term *remineralisation* does not chemically equate to remineralisation.

Premolars of the upper jaw had the highest fraction of progressed lesions, followed by molars in the lower and upper jaw. The reason for the high rate of caries progression in premolars in the upper jaw can only be speculated. However, compared to premolars in the lower jaw, they are farther away from salivary ducts and probably have less cleaning effect from the tongue. Furthermore, the anatomy of the first premolar of the upper jaw, with a distinct cavity on the mesial surface, may increase plaque retention.

### Strength and limitations

This study followed a relatively large, well-defined cohort (N = 584) for 10 years, which is a significant strength as it enabled the observation of long-term trends and the establishment of temporal relationships between risk factors and outcomes. The cohort included both men and women in equal proportions, enhancing the generalisability of the findings to both sexes. The examinations and caries registrations were standardised with thorough training and calibration of the observers. The use of standardised, high-resolution, clinical photos allowed reevaluation, optimal viewing conditions, less time pressure, and discussions upon uncertainty when performing the caries registration.

The study also has some limitations. Some of the differences observed between ages 17 and 27 may be due to systematic differences in caries registration between the 2 observers involved in the recordings. However, the similarity in distribution between various tooth groups and surfaces at the 2 observation points suggests that the calibration of the observers was adequate.

We merged grade 1 and 2 enamel caries, which caused some loss of nuances in the analyses (eg, those who had enamel caries lesions progressing from grade 1 to grade 2 were not identified). We chose to do this because different projections of x-rays between studies, erosions, and other tooth wear may affect interpretations and cause high levels of uncertainty, especially when assessing caries progression within the enamel.

Reasons for missing teeth were not recorded. We assumed that most missing teeth were not due to caries and therefore excluded them from the analyses, which will have caused some underestimation of caries experience in the population.

The study cohort experienced differential dropout between ages 17 and 27, with relatively higher attrition among male, participants attending vocational USS programs and persons with higher dentin caries prevalence. We found that sex, SES, and caries experience were all significantly associated with enamel lesion rate and progression; thus, the dropout may have introduced selection bias and compromised the generalisability of the results. Whereas men had somewhat lower rates of enamel caries and progression, lower SES and higher caries experience were both associated with higher rates, making it difficult to estimate the effect of the dropout on the results.

The mixed-effects NB model effectively captured the overall trend in enamel caries lesions, as demonstrated by the close agreement between the predicted mean and the observed mean. The fixed effects were robust, and the inclusion of random intercepts was well justified, accounting for variability at the individual level. However, some limitations were identified, including underprediction for extreme values, which may indicate that the model does not fully capture the variability in individuals with very high lesion counts. While the main risk factors were tested, there is always the possibility of unmeasured factors that could introduce residual confounding. Additionally, while residual dependence was observed, it was low and within an acceptable range, suggesting that the model performed well overall. The observed residual dependence may indicate the influence of unmeasured factors, such as genetic predispositions, saliva pH and composition, or fluoride exposure. In Norway, toothbrushing with fluoridated toothpaste twice a day is the general recommendation, and almost all available toothpastes contain fluoride. Toothbrushing frequency therefore also indicates fluoride exposure. Extra supplements, such as fluoridated mouthwashes or tablets, are recommended based on an individualised caries risk assessment. Thus, including such variables in the models could introduce confounding by indication. We had to limit the number of explanatory variables in the statistical models due to the moderate cohort size to avoid overfitting.

To analyse associations between risk factors and enamel caries progression, we employed 3 regression models, each providing complementary insights. The NB and ZINB models showed differences in identifying risk factors, as discussed in Supplementary Appendices 5 and 7.

## Conclusions

Enamel caries was almost ubiquitous, and the number of dentin lesions increased from adolescence to adulthood. Almost 20% of enamel caries lesions progressed to dentin caries, most frequently on premolars of the upper jaw and on proximal surfaces. Well-established risk factors of caries were associated with the number and progression of enamel caries lesions, supporting a focus on oral hygiene and restriction of sugar as efficient prophylactic strategies. Extra attention should be given to individuals with moderate or poor general health and those having medium or low SES. The high fraction of enamel caries lesions that did not progress lends strong support to the nonoperative treatment of enamel caries and implementation of preventive measures.

## Author contributions

Anne-Sofie Furberg participated in planning and conducting the Fit Future cohort study, and Elin Hadler-Olsen participated in conception and data collection for the current substudy. Data preparation, data analysis, and drafting of the manuscript were performed by Elin Hadler-Olsen and Natalia Petrenya. All authors contributed to the study design, provided a critical revision of the manuscript, and read and approved the final version.

## Declaration of generative AI and AI-assisted technologies in the manuscript preparation process

During the preparation of this work, the authors used Chat GPT-4.0 at UiT the Arctic University of Norway for proofreading and language editing. After using this tool, the authors reviewed and edited the content as needed and take full responsibility for the content of the published article.

## Funding

The authors declare that no funds, grants, or other support were received during the preparation of this manuscript.

## Availability of data and materials

The data that support the findings of this study are available from the Fit Futures study, but restrictions apply to the availability of these data, which were used under license for the current study and so are not publicly available. Data are, however, available from the authors upon reasonable request and with permission of the Fit Futures study: Fit Futures | UiT.

## Conflict of interest

None disclosed.

## Appendix Supplementary materials

Image, application 1

Image, application 2

## Footnotes

Supplementary material associated with this article can be found in the online version at doi:10.1016/j.identj.2026.109739.
