# Supplementary material for: Enamel Caries in Young Adults and Progression From Adolescence: Fit Futures, a Longitudinal Cohort Study
Source: Int Dent J. 2026 Jul 15;76(5):109739. doi: 10.1016/j.identj.2026.109739 (PMC13383994; doi:10.1016/j.identj.2026.109739)
Supplement: Supplementary file 1 [file mmc1.docx]

**Supplementary file**

**«Enamel caries in young adults and progression from adolescence – Fit Futures a longitudinal cohort study”**

**Appendix 1 Detailed assessment of six dietary determinants in sensitivity analyses**

**Characteristics of dietary variables**

Six cariogenic dietary variables assessed at ages 17 and 27 were included in this study (Table S1). The variable intake of sports drinks with sugar was not used, as intake was assessed only at age 27, when 93% of the participants reported rarely/never consuming them (<1 glass/week).

**Table S1** Characteristics of dietary variables at ages 17 and 27 years

|  | 17 years  n % | | 27 years  n % | |
| --- | --- | --- | --- | --- |
| **How often do you usually eat:**  **Sweets (e.g., chocolate, candy)** |  |  |  |  |
| Rarely/never | 42 | 7.3 | 31 | 5.6 |
| 1-3 times/month | 117 | 20.2 | 143 | 26.0 |
| 1-3 times/week | 337 | 58.3 | 312 | 56.6 |
| 4-6 times/week | 68 | 11.8 | 54 | 9.8 |
| Every day | 14 | 2.4 | 11 | 2.0 |
| **Fruits** |  |  |  |  |
| Rarely/never | 23 | 4.0 | 14 | 2.5 |
| 1-3 times/month | 61 | 10.5 | 85 | 15.3 |
| 1-3 times/week | 171 | 29.5 | 206 | 37.2 |
| 4-6 times/week | 122 | 21.1 | 146 | 26.4 |
| Every day | 202 | 34.9 | 103 | 18.6 |
| **Snacks (e.g., chips, biscuits, cakes, buns) 17 years** |  |  |  |  |
| Rarely/never | 27 | 4.7 |  |  |
| 1-3 times/month | 169 | 29.2 |  |  |
| 1-3 times/week | 302 | 52.2 |  |  |
| 4-6 times/week | 71 | 12.3 |  |  |
| Every day | 10 | 1.7 |  |  |
| **Sweet snacks (e.g., biscuits, cakes, buns) 27 years** |  |  |  |  |
| Rarely/never |  |  | 90 | 16.3 |
| 1-3 times/month |  |  | 276 | 49.9 |
| 1-3 times/week |  |  | 178 | 32.2 |
| 4-6 times/week |  |  | 8 | 1.4 |
| Every day |  |  | 1 | 0.2 |
| **Salty snacks (e.g., chips, nuts) 27 years** |  |  |  |  |
| Rarely/never |  |  | 38 | 6.9 |
| 1-3 times/month |  |  | 212 | 38.3 |
| 1-3 times/week |  |  | 288 | 52.1 |
| 4-6 times/week |  |  | 15 | 2.7 |
| Every day |  |  | 0 | 0.0 |
| **Harmonized ^1^ snacks** |  |  |  |  |
| Rarely/never | 27 | 4.7 | 17 | 3.1 |
| 1-3 times/month | 169 | 29.2 | 190 | 34.4 |
| 1-3 times/week | 302 | 52.2 | 325 | 58.8 |
| ≥4 times/week | 81 | 14.0 | 21 | 3.8 |
| **How often do you usually drink:** |  |  |  |  |
| **Fruit juice (100 % pure)** |  |  |  |  |
| Rarely/never | 102 | 17.7 | 307 | 55.5 |
| 1-6 glasses/week | 276 | 47.8 | 218 | 39.4 |
| 1 glass/day | 117 | 20.3 | 23 | 4.2 |
| ≥ 2 glasses/day | 69 | 12.0 | 4 | 0.7 |
| ≥ 4 glasses/day | 13 | 2.3 | 1 | 0.2 |
| **Soft drinks with sugar** |  |  |  |  |
| Rarely/never | 136 | 23.6 | 403 | 73.5 |
| 1-6 glasses/week | 320 | 55.6 | 125 | 22.8 |
| 1 glass/day | 59 | 10.2 | 12 | 2.2 |
| ≥ 2 glasses/day | 61 | 10.6 | 8 | 1.5 |
| **Sugar sweetened fruit drinks (FF1: diluted syrups; FF3: diluted syrups, icetea)** |  |  |  |  |
| Rarely/never | 366 | 63.3 | 473 | 85.7 |
| 1-6 glasses/week | 161 | 27.9 | 71 | 12.9 |
| 1 glass/day | 29 | 5.0 | 6 | 1.1 |
| ≥ 2 glasses/day | 20 | 3.5 | 2 | 0.4 |
| ≥ 4 glasses/day | 2 | 0.3 | 0 | 0.0 |

**^1^** Harmonization of snack consumption variables

Snack consumption data were collected at two points: at age 17 (FF1) and age 27 (FF3). At FF1, snack consumption was assessed using a single variable (SNACKS_FF1), which captured the frequency of consuming both sweet and salty snacks combined. At FF3, snack consumption was assessed using two separate variables: sweet snacks (SWEET_SNACKS_FF3) and salty snacks (SALTY_SNACKS_FF3). To harmonize these variables across time points, a new variable (combined_snacks_FF3) was created for FF3 by taking the higher frequency of consumption between sweet and salty snacks for each individual. If the frequencies of sweet and salty snacks were equal, the shared value was assigned to the combined variable. A final harmonized variable (harmonized_snacks) was created to represent snack consumption across both time points. For FF1, the original SNACKS_FF1 variable was used, while for FF3, the combined_snacks_FF3 variable was assigned. The harmonized variable had the following categories: 0 "rarely/never", 1 "1–3 times/month", 2 "1–3 times/week", 3 " ≥4 times/week" and was used in the mixed-effects models to analyze its association with enamel caries lesions.

**Justification for including soft drinks in the final model**

When deciding which dietary variables to include in the final mixed-effects models, we considered the research question, theoretical framework, existing knowledge, the variability of dietary variables across time points (i.e., between FF1 and FF3), and the results of statistical modeling. We examined the relationship between dietary variables and the number of enamel caries lesions at ages 17 and 27. All six dietary variables were tested separately (Table S2: univariable models) and simultaneously (Table S2: multivariable (mutually adjusted model)) since no strong multicollinearity was observed, as indicated by Spearman correlation analysis and the VIF test (data not shown).

**Table S2** Univariable and mutually adjusted multivariable mixed‑effects models for the six dietary factors

|  | **Univariable** | | | | **Multivariable**  **(mutually adjusted model)** | | | |
| --- | --- | --- | --- | --- | --- | --- | --- | --- |
| **Number of enamel caries lesions**  **(ages 17 and 27)** | IRR | (95% CI) | | p | IRR | (95% CI) | | p |
| Factors |  |  |  |  |  |  |  |  |
| **Soft drinks** |  |  |  |  |  |  |  |  |
| 1-6 glasses/week | **1.22** | **(1.13–1.31)** | | **<.001** | **1.17** | **(1.07–1.27)** | | **<.001** |
| 1 glass/day | **1.30** | **(1.12–1.50)** | | **<.001** | **1.19** | **(1.01–1.40)** | | **.043** |
| ≥ 2 glasses/day | **1.46** | **(1.29–1.66)** | | **<.001** | **1.31** | **(1.12–1.52)** | | **.001** |
| **Fruit juice** |  |  |  |  |  |  |  |  |
| 1-6 glasses/week | **1.19** | **(1.10–1.28)** | | **<.001** | 1.10 | (1.01–1.20) | | .022 |
| 1 glass/day | **1.22** | **(1.08–1.36)** | | **.001** | 1.07 | (0.94–1.21) | | .330 |
| ≥ 2 glasses/day | **1.28** | **(1.11–1.47)** | | **.001** | 1.09 | (0.92–1.29) | | .308 |
| **Fruit drinks** |  |  |  |  |  |  |  |  |
| 1-6 glasses/week | **1.13** | **(1.04–1.23)** | | **.003** | 1.03 | (0.94–1.13) | | .498 |
| ≥1 glasses/day | **1.27** | **(1.13–1.44)** | | **<.001** | 1.08 | (0.93–1.25) | | .312 |
| **Sweets** |  |  |  |  |  |  |  |  |
| 1-3 times/month | 0.85 | (0.72–1.01) | | .067 | 0.84 | (0.69–1.02) | | .072 |
| 1-3 times/week | 0.87 | (0.73–1.02) | | .092 | 0.83 | (0.69–1.01) | | .066 |
| 4-6 times/week | 1.01 | (0.83–1.23) | | .945 | 0.88 | (0.70–1.11) | | .289 |
| Every day | 0.90 | (0.68–1.20) | | .477 | 0.79 | (0.57–1.09) | | .147 |
| **Fruits** |  |  |  |  |  |  |  |  |
| 1-3 times/month | 0.87 | (0.69–1.10) | | .246 | 0.92 | (0.73–1.16) | | .485 |
| 1-3 times/week | 0.82 | (0.66–1.03) | | .095 | 0.88 | (0.70–1.10) | | .254 |
| 4-6 times/week | 0.82 | (0.65–1.03) | | .084 | 0.89 | (0.70–1.12) | | .302 |
| Every day | 0.83 | (0.66–1.04) | | .108 | 0.87 | (0.69–1.10) | | .247 |
| **Snacks** |  |  |  |  |  |  |  |  |
| 1-3 times/month | 1.02 | (0.83–1.24) | | .868 | 1.11 | (0.88–1.40) | | .386 |
| 1-3 times/week | 1.00 | (0.82–1.22) | | .982 | 1.08 | (0.85–1.37) | | .545 |
| ≥4 times/week | **1.27** | **(1.02**–**1.58)** | | **.036** | 1.19 | (0.90–1.58) | | .217 |

Numbers in bold indicate statistically significant associations. Abbreviation: Incidence Rate Ratio (IRR), Confidence interval (CI).

In the univariable models, soft drink, fruit juice, fruit drinks (sweetened), and snacks showed significant positive associations with the number of enamel caries lesions, while fruits and sweets did not. However, in the multivariable (mutually adjusted) models, only soft drinks remained consistently significant and exhibited a dose-response relationship (Table S2). All six dietary variables were tested both individually and jointly in models that adjusted for other risk factors (sex, SES, general health, toothbrushing, dental visits, and D_D_FS; Table S3, Models 1–7). Soft drinks remained significantly associated with enamel caries after adjustment for sex, SES, general health, toothbrushing, dental visits, and D_D_FS (Table S3, Model 1). The association remained significant when all dietary variables were included (Table S3, Model 7), supporting soft drinks inclusion in the final model.

To simplify the final model, avoid overfitting, and retain the most relevant variable, we focused on soft drink intake, which emerged as a consistent risk factor and showed clear variability between ages 17 and 27. The soft drink intake variable was modified to separate between- and within-individual effects in the final model.

**Table S3 Multivariable mixed‑effects models for dietary and non‑dietary factors**

**Outcome: number of enamel caries lesions (ages 17 and 27)**

|  | **Model 1 Soft drinks** | |  | **Model 2 Fruit juice** | |  | **Model 3 Fruit drinks** | |  | **Model 4 Sweets** | |  | **Model 5 Fruits** | |  | **Model 6 Snacks** | |  | **Model 7 All dietary factors** | | |
| --- | --- | --- | --- | --- | --- | --- | --- | --- | --- | --- | --- | --- | --- | --- | --- | --- | --- | --- | --- | --- | --- |
|  | IRR | 95% CI | p | IRR | 95% CI | p | IRR | (95% CI) | p | IRR | (95% CI) | p | IRR | (95% CI) | p | IRR | (95% CI) | p | IRR | (95% CI) | p |
| **Factors**  **Sex** |  |  |  |  |  |  |  |  |  |  |  |  |  |  |  |  |  |  |  |  |  |
| Female |  |  |  |  |  |  |  |  |  |  |  |  |  |  |  |  |  |  |  |  |  |
| Male | **0.87** | **(0.79–0.96)** | **.005** | 0.91 | (0.83–1.00) | .063 | 0.91 | (0.83–1.00) | .051 | 0.91 | (0.83–1.00) | .057 | 0.93 | (0.84–1.02) | .126 | 0.92 | (0.83–1.01) | .076 | **0.87** | **(0.79–0.97)** | **.008** |
| **SES** |  |  |  |  |  |  |  |  |  |  |  |  |  |  |  |  |  |  |  |  |  |
| Medium/low | 1.00 | (1.00–1.00) |  | 1.00 | (1.00–1.00) |  | 1.00 | (1.00–1.00) |  | 1.00 | (1.00–1.00) |  | 1.00 | (1.00–1.00) |  | 1.00 | (1.00–1.00) |  | 1.00 | (1.00–1.00) |  |
| High | **0.89** | **(0.79–0.99)** | **.039** | **0.88** | **(0.78–0.98)** | **.022** | **0.86** | **(0.77–0.96)** | **.008** | **0.86** | **(0.77–0.96)** | **.005** | **0.85** | **(0.76–0.95)** | **.005** | **0.87** | **(0.78–0.97)** | **.010** | 0.89 | (0.80–1.00) | .053 |
| **General health** |  |  |  |  |  |  |  |  |  |  |  |  |  |  |  |  |  |  |  |  |  |
| Moderate/poor | **1.09** | **(1.00–1.19)** | **.048** | **1.11** | **(1.02–1.21)** | **.017** | **1.09** | **(1.00–1.20)** | **.045** | **1.10** | **(1.01–1.20)** | **.032** | **1.10** | **(1.01–1.21)** | **.028** | 1.09 | (1.00–1.19) | .054 | **1.10** | **(1.01–1.20)** | **.033** |
| Good | 1.00 | (1.00–1.00) |  | 1.00 | (1.00–1.00) |  | 1.00 | (1.00–1.00) |  | 1.00 | (1.00–1.00) |  | 1.00 | (1.00–1.00) |  | 1.00 | (1.00–1.00) |  | 1.00 | (1.00–1.00) |  |
| **Toothbrushing** |  |  |  |  |  |  |  |  |  |  |  |  |  |  |  |  |  |  |  |  |  |
| < 2/day | **1.13** | **(1.04–1.24)** | **.005** | **1.15** | **(1.05–1.26)** | **.002** | 1.16 | (1.06–1.27) | .001 | **1.18** | **(1.07–1.29)** | **<.001** | **1.17** | **(1.07–1.28)** | **<.001** | **1.17** | **(1.07–1.28)** | **.001** | **1.12** | **(1.02–1.22)** | **.014** |
| ≥ 2/day | 1.00 | (1.00–1.00) |  | 1.00 | (1.00–1.00) |  | 1.00 | (1.00–1.00) |  | 1.00 | (1.00–1.00) |  | 1.00 | (1.00–1.00) |  | 1.00 | (1.00–1.00) |  | 1.00 | (1.00–1.00) |  |
| **Dental visits** |  |  |  |  |  |  |  |  |  |  |  |  |  |  |  |  |  |  |  |  |  |
| ≥ 1/2 year | 1.00 | (1.00–1.00) |  | 1.00 | (1.00–1.00) |  | 1.00 | (1.00–1.00) |  | 1.00 | (1.00–1.00) |  | 1.00 | (1.00–1.00) |  | 1.00 | (1.00–1.00) |  | 1.00 | (1.00–1.00) |  |
| < 1/2 year | 1.02 | (0.91–1.15) | .685 | 1.05 | (0.93–1.18) | .416 | 1.03 | (0.91–1.15) | .651 | 1.03 | (0.92–1.16) | .592 | 1.02 | (0.91–1.15) | .740 | 1.02 | (0.90–1.15) | .764 | 1.05 | (0.93–1.18) | .410 |
| Acute/never | **1.14** | **(1.02–1.27)** | **.017** | **1.17** | **(1.05–1.31)** | **.004** | **1.16** | **(1.04–1.30)** | **.009** | **1.15** | **(1.03–1.28)** | **.011** | **1.16** | **(1.04–1.29)** | **.010** | **1.15** | **(1.03–1.28)** | **.014** | **1.16** | **(1.04–1.30)** | **.007** |
| **D_D_FS spline** |  |  |  |  |  |  |  |  |  |  |  |  |  |  |  |  |  |  |  |  |  |
| D_D_FS (spline1) | **1.03** | **(1.02–1.05)** | **<.001** | **1.04** | **(1.02–1.05)** | **<.001** | **1.03** | **(1.02–1.05)** | **<.001** | **1.03** | **(1.02–1.05)** | **<.001** | **1.03** | **(1.02–1.05)** | **<.001** | **1.03** | **(1.02–1.05)** | **<.001** | **1.04** | **(1.03–1.05)** | **<.001** |
| D_D_FS (spline 2) | 1.01 | (1.00–1.01) | .055 | 1.01 | (1.00–1.01) | .081 | 1.00 | (1.00–1.01) | .195 | 1.00 | (1.00–1.01) | .171 | 1.00 | (1.00–1.01) | .279 | 1.00 | (1.00–1.01) | .198 | **1.01** | **(1.00–1.01)** | **.020** |
| **Soft drinks** |  |  |  |  |  |  |  |  |  |  |  |  |  |  |  |  |  |  |  |  |  |
| Rarely/never | 1.00 | (1.00–1.00) |  |  |  |  |  |  |  |  |  |  |  |  |  |  |  |  | 1.00 | (1.00–1.00) |  |
| 1-6 glasses/week | **1.23** | **(1.14–1.33)** | **<.001** |  |  |  |  |  |  |  |  |  |  |  |  |  |  |  | **1.19** | **(1.09–1.30)** | **<.001** |
| 1 glass/day | **1.37** | **(1.18–1.60)** | **<.001** |  |  |  |  |  |  |  |  |  |  |  |  |  |  |  | **1.26** | **(1.07–1.49)** | **.007** |
| ≥ 2 glasses/day | **1.44** | **(1.26–1.66)** | **<.001** |  |  |  |  |  |  |  |  |  |  |  |  |  |  |  | **1.28** | **(1.09–1.51)** | **.003** |
| **Fruit juice** |  |  |  |  |  |  |  |  |  |  |  |  |  |  |  |  |  |  |  |  |  |
| Rarely/never |  |  |  | 1.00 | (1.00–1.00) |  |  |  |  |  |  |  |  |  |  |  |  |  | 1.00 | (1.00–1.00) |  |
| 1-6 glasses/week |  |  |  | **1.20** | **(1.10–1.29)** | **<.001** |  |  |  |  |  |  |  |  |  |  |  |  | **1.12** | **(1.03–1.22)** | **.009** |
| 1 glass/day |  |  |  | **1.28** | **(1.13–1.44)** | **<.001** |  |  |  |  |  |  |  |  |  |  |  |  | 1.11 | (0.98–1.27) | .104 |
| ≥ 2 glasses/day |  |  |  | **1.37** | **(1.19–1.58)** | **<.001** |  |  |  |  |  |  |  |  |  |  |  |  | 1.18 | (0.99–1.40) | .058 |
| **Fruit drinks** |  |  |  |  |  |  |  |  |  |  |  |  |  |  |  |  |  |  |  |  |  |
| Rarely/never |  |  |  |  |  |  | 1.00 | (1.00–1.00) |  |  |  |  |  |  |  |  |  |  | 1.00 | (1.00–1.00) |  |
| 1-6 glasses/week |  |  |  |  |  |  | **1.12** | **(1.02–1.22)** | **.016** |  |  |  |  |  |  |  |  |  | 1.02 | (0.92–1.12) | .764 |
| ≥1 glasses/day |  |  |  |  |  |  | **1.30** | **(1.14–1.48)** | **<.001** |  |  |  |  |  |  |  |  |  | 1.11 | (0.95–1.30) | .191 |
| **Sweets** |  |  |  |  |  |  |  |  |  |  |  |  |  |  |  |  |  |  |  |  |  |
| Rarely/never |  |  |  |  |  |  |  |  |  | 1.00 | (1.00–1.00) |  |  |  |  |  |  |  | 1.00 | (1.00–1.00) |  |
| 1-3 times/month |  |  |  |  |  |  |  |  |  | **0.80** | **(0.67–0.95)** | **.012** |  |  |  |  |  |  | **0.78** | **(0.64–0.94)** | **.011** |
| 1-3 times/week |  |  |  |  |  |  |  |  |  | **0.83** | **(0.70–0.98)** | **.033** |  |  |  |  |  |  | **0.77** | **(0.63–0.94)** | **.009** |
| 4-6 times/week |  |  |  |  |  |  |  |  |  | 0.93 | (0.76–1.14) | .464 |  |  |  |  |  |  | 0.80 | (0.63–1.01) | .058 |
| every day |  |  |  |  |  |  |  |  |  | 0.78 | (0.58–1.04) | .088 |  |  |  |  |  |  | **0.66** | **(0.47–0.92)** | **.015** |
| **Fruits** |  |  |  |  |  |  |  |  |  |  |  |  |  |  |  |  |  |  |  |  |  |
| Rarely/never |  |  |  |  |  |  |  |  |  |  |  |  | 1.00 | (1.00–1.00) |  |  |  |  | 1.00 | (1.00–1.00) |  |
| 1-3 times/month |  |  |  |  |  |  |  |  |  |  |  |  | 0.87 | (0.69–1.10) | .254 |  |  |  | 0.90 | (0.71–1.13) | .366 |
| 1-3 times/week |  |  |  |  |  |  |  |  |  |  |  |  | 0.86 | (0.68–1.08) | .198 |  |  |  | 0.89 | (0.71–1.11) | .297 |
| 4-6 times/week |  |  |  |  |  |  |  |  |  |  |  |  | 0.88 | (0.70–1.12) | .302 |  |  |  | 0.92 | (0.73–1.16) | .469 |
| every day |  |  |  |  |  |  |  |  |  |  |  |  | 0.93 | (0.74–1.18) | .557 |  |  |  | 0.94 | (0.74–1.19) | .616 |
| **Snacks** |  |  |  |  |  |  |  |  |  |  |  |  |  |  |  |  |  |  |  |  |  |
| Rarely/never |  |  |  |  |  |  |  |  |  |  |  |  |  |  |  | 1.00 | (1.00–1.00) |  | 1.00 | (1.00–1.00) |  |
| 1-3 times/month |  |  |  |  |  |  |  |  |  |  |  |  |  |  |  | 1.04 | (0.85–1.29) | .682 | 1.19 | (0.94–1.51) | .140 |
| 1-3 times/week |  |  |  |  |  |  |  |  |  |  |  |  |  |  |  | 1.04 | (0.84–1.28) | .742 | 1.17 | (0.92–1.49) | .194 |
| ≥ 4 times/week |  |  |  |  |  |  |  |  |  |  |  |  |  |  |  | **1.27** | **(1.00–1.60)** | **.046** | 1.29 | (0.97–1.73) | .081 |
| / |  |  |  |  |  |  |  |  |  |  |  |  |  |  |  |  |  |  |  |  |  |
| lnalpha | 0.20 | (0.15–0.25) | <.001 | 0.19 | (0.15–0.25) | <.001 | 0.20 | (0.16–0.26) | <.001 | 0.21 | (0.16–0.27) | <.001 | 0.21 | (0.16–0.27) | <.001 | 0.21 | (0.16–0.26) | <.001 | 0.18 | (0.14–0.24) | <.001 |
| var(_cons[id]) | 1.14 | (1.08–1.19) | <.001 | 1.15 | (1.09–1.21) | <.001 | 1.14 | (1.08–1.20) | <.001 | 1.13 | (1.07–1.19) | <.001 | 1.13 | (1.08–1.19) | <.001 | 1.14 | (1.08–1.19) | <.001 | 1.14 | (1.09–1.19) | <.001 |
| N |  | 1092 |  |  | 1099 |  |  | 1098 |  |  | 1098 |  |  | 1101 |  |  | 1101 |  |  | 1086 |  |
|  |  |  |  |  |  |  |  |  |  |  |  |  |  |  |  |  |  |  |  |  |  |
| Abbreviation: Dentin caries decayed and filled surfaces (D_D_FS). Incidence Rate Ratio (IRR). Confidence interval (CI). Socioeconomic status (SES). | | | | | | | | | | | | |  |  |  |  |  |  |  |  |  |
| Numbers in bold indicate statistically significant associations. | | | | |  |  |  |  |  |  |  |  |  |  |  |  |  |  |  |  |  |

**Appendix 2**

**Table S4** Mixed-effects multivariable model excluding dental visit variable in relation tonumber of enamel caries lesions (ages 17 to 27)

|  | **Multivariable model (n=582)** | |
| --- | --- | --- |
| **Number of enamel caries lesions**  **(ages 17 and 27)** | IRR (95% CI) | p |
| Factors |  |  |
| **Sex** |  |  |
| Women | Reference |  |
| Men | **0.89 (0.81–0.99)** | **.026** |
| **SES ^1^** |  |  |
| High | Reference |  |
| Medium/low | **1.14 (1.01–1.27)** | **.027** |
| **General health** |  |  |
| Good | Reference |  |
| Moderate/poor | **1.10 (1.01–1.19)** | **.029** |
| **Toothbrushing** |  |  |
| ≥2/day | Reference |  |
| <2/day | **1.15 (1.06–1.26)** | **.001** |
| **D_D_FS ^3^ spline (knot at D**_D_**FS=11)** |  |  |
| D_D_FS (spline 1) | **1.04 (1.02–1.05)** | **<.001** |
| D_D_FS (spline 2) | 1.01 (1.00–1.01) | .095 |
| **Soft drinks (between-effect) ^2^** |  |  |
| Rarely/never | Reference |  |
| 1-6 glasses/week | 1.12 (0.98–1.29) | .104 |
| 1 glass/day | 1.31 (1.00–1.72) | .051 |
| ≥2 glasses/day | **1.47 (1.15–1.86)** | **.002** |
| **Soft drinks (within-effect) ^2^** |  |  |
| Rarely/never | Reference |  |
| 1-6 glasses/week | **1.31 (1.19–1.44)** | **<.001** |
| 1 glass/day | **1.36 (1.15–1.61)** | **<.001** |
| ≥2 glasses/day | **1.44 (1.21–1.72)** | **<.001** |

^1^ SES was computed based on the variables upper secondary school program, education and financial situation, where having a combination of academic upper secondary school program, higher education of at least 4 years and reporting a good financial situation were defined as high SES, and all other combinations as medium/low SES. ^2^ Between-individual effect: modeled by taking the average soft drink intake for each person across all time points. Within-individual effect: modeled by calculating the deviation of each observation from that individual's average at each time point. ^3^D_D_FS index estimates sum of decayed-filled surfaces or permanent restorations. Numbers in bold indicate statistically significant associations.

Abbreviation: Socioeconomic status (SES), Dentin caries decayed and filled surfaces (D_D_FS), Incidence Rate Ratio (IRR), Confidence interval (CI).

**Appendix 3**

**Justification of zero-inflated negative binomial (ZINB) model and the choice of variables for count and inflation part**

Logistic regression models the probability of a binary outcome (i.e., presence or absence of enamel caries lesions), whereas the zero-inflation component of the ZINB model specifically addresses the probability of belonging to the "not-at-risk" group (structural zeros). While related, these concepts are distinct. The ZINB model is particularly suited for handling excess zeros, making it more appropriate for datasets with a notable proportion of zeros, such as the 36% observed here. This proportion suggests the need for a zero-inflated approach, as the excess zeros are likely generated by a mechanism separate from the count data. Prior logistic regression results can guide variable selection for the inflation part of the ZINB model, but these variables should be theoretically justified. Protective behaviors (e.g., frequent toothbrushing and higher education) or inherent resistance (e.g., genetic factors) are more likely to influence the " not-at-risk " group, while risk factors (e.g., diet and health conditions) are more likely to affect the count component.

**Appendix 4**

**Table S5** Crude association between baseline risk factors assessed at age 17 and enamel caries lesion progression

| **Number of progressed enamel caries lesions** | **Univariable models** | | | |
| --- | --- | --- | --- | --- |
|  | **Logistic** | | **Negative binomial** | |
| Factors | OR (95% CI) | p | IRR (95% CI) | p |
| **Sex** |  |  |  |  |
| Women | Reference |  | Reference |  |
| Men | 1.14 (0.81–1.60) | .457 | 0.98 (0.80–1.22) | .876 |
| **General health** |  |  |  |  |
| Good | Reference |  | Reference |  |
| Moderate/poor | **1.73 (1.15–2.61)** | **.009** | **1.51 (1.19–1.91)** | **.001** |
| **Soft drinks** |  |  |  |  |
| Rarely | Reference |  | Reference |  |
| 1-6 glasses/week | 1.38 (0.92–2.08) | .119 | 1.20 (0.92–1.57) | .170 |
| ≥1 glass/day | **2.20 (1.29–3.74)** | **.004** | **1.57 (1.15–2.16)** | **.005** |
| **USS program** |  |  |  |  |
| USS academic/sport | Reference |  | Reference |  |
| USS vocational | **2.04 (1.43–2.91)** | **<.001** | **1.73 (1.40–2.13)** | **<.001** |
| **Toothbrushing** |  |  |  |  |
| <2/day | Reference |  | Reference |  |
| ≥2/day | **0.37 (0.25–0.55)** | **<.001** | **0.52 (0.42–0.64)** | **<.001** |

Numbers in bold indicate statistically significant associations.

Abbreviation: Odds Ratio (OR), Incidence Rate Ratio (IRR), Confidence interval (CI), upper secondary school (USS).

**Appendix 5**

**A comparison of the NB and ZINB model fits and the interpretation of the model results for factors assessed at age 17 (See Table 5 in the main manuscript).**

The NB model demonstrated slightly better overall fit metrics, with lower AIC (2259.27 vs. 2272.63), BIC (2294.05 vs. 2311.76), and a higher log-likelihood (-1121.633 vs. -1127.317) compared to the ZINB model. However, the Vuong test (p = .001) indicated that the ZINB model was significantly better at addressing zero-inflation in the dataset.

Soft drink intake was identified as a significant risk factor for enamel caries lesion progression, particularly among participants with non-zero outcomes. The ZINB model's ability to account for excess zeros allowed it to detect this relationship, which was attenuated in the NB model. By distinguishing between structural zeros (e.g., due to protective factors like frequent toothbrushing) and zeros unrelated to soft drink intake, the ZINB model provided additional insights into the data.

In the zero-inflation part, attending an academic or sports upper secondary school program was a factor significantly influencing the likelihood of belonging to the "not-at-risk" group. Toothbrushing ≥2 times per day was also associated with a significantly higher likelihood of being in the "not-at-risk" group. All the coefficients were statistically significant (p-values < 0.05), indicating that the effects of education and toothbrushing are unlikely to be due to random chance and that they increase the odds of a zero outcome. However, the large range between the lower and upper confidence interval limits reflects uncertainty about the exact magnitude of the effect of toothbrushing. This highlights the need to interpret the effect size with caution.

**Appendix 6**

**Table S6** Crude association between risk factors assessed at age 27 and enamel caries lesions progression

| **Number of progressed enamel caries lesions** | **Univariable models** | | | |
| --- | --- | --- | --- | --- |
|  | **Logistic** | | **Negative binomial** | |
| Factors | OR (95% CI) | p | IRR (95% CI) | p |
| **Sex** |  |  |  |  |
| Women | Reference |  | Reference |  |
| Men | 1.14 (0.81–1.60) | .457 | 0.98 (0.80–1.22) | .876 |
| **General health** |  |  |  |  |
| Good | Reference |  | Reference |  |
| Moderate/poor | 1.50 (1.00–2.26) | .053 | **1.35 (1.05–1.72)** | **.017** |
| **Soft drinks** |  |  |  |  |
| Rarely | Reference |  | Reference |  |
| ≥1/week | **1.68 (1.11–2.54)** | **.015** | 1.24 (0.97–1.58) | .092 |
| **Dental visits** |  |  |  |  |
| ≥1/2 year | Reference |  | Reference |  |
| <1/2 year | 0.84 (0.53–1.33) | .468 | 0.80 (0.60–1.08) | .146 |
| Acute/never | 1.06 (0.70–1.62) | .773 | 0.98 (0.76–1.28) | .900 |
| **Education** |  |  |  |  |
| Higher ≥4 years | Reference |  | Reference |  |
| Secondary school | **4.55 (1.78–11.60)** | **.002** | **2.57 (1.63–4.06)** | **<.001** |
| USS academic/sport | **2.24 (1.36–3.68)** | **.001** | **1.88 (1.38–2.56)** | **<.001** |
| USS vocational | **2.47 (1.42–4.30)** | **.001** | **1.99 (1.43–2.77)** | **<.001** |
| Higher <4 years | **1.80 (1.14–2.85)** | **.012** | **1.47 (1.09–1.97)** | **.011** |
| **Toothbrushing** |  |  |  |  |
| <2/day | Reference |  | Reference |  |
| ≥2/day | **0.43 (0.28–0.66)** | **<.001** | **0.60 (0.48–0.76)** | **<.001** |

Numbers in bold indicate statistically significant associations.

Abbreviation: Odds Ratio (OR), Incidence Rate Ratio (IRR), Confidence interval (CI), upper secondary school (USS)

**Appendix 7**

**A comparison of the NB and ZINB model fits and the interpretation of the model results for factors assessed at age 27 (See Table 6 in the main manuscript)**

The ZINB model demonstrated a higher log-likelihood (-1083.993) compared to the NB model (-1101.527), indicating a better fit to the data. While the NB model had slightly lower AIC (2182.13 vs. 2184.59) and BIC (2233.8 vs. 2240.58) values, the differences were minimal, suggesting that both models fit the data similarly well. The ZINB model's ability to handle excess zeros makes it more suitable for datasets with a high proportion of zeros. The Vuong test (p = .002) confirmed that the ZINB model was significantly better at addressing zero-inflation compared to the NB model.

The ZINB model effectively captured the relationship between poorer general health and increased enamel lesion progression, showing a 29% higher number of progressed lesions among participants with poorer health (p = .027). This highlights the ZINB model's strength in accounting for zero-inflation and supports its use when excess zeros are present. Both models are reasonable choices and provide a detailed understanding of relationships.

In the zero-inflation part, the trend of higher education as a protective factor, observed in a similar model based on baseline data, was confirmed. Additionally, toothbrushing ≥2 times per day was associated with a borderline significant higher likelihood of belonging to the "not-at-risk" group (p = .065). However, in this model, the large range between the lower and upper confidence interval for some education groups and toothbrushing reflects uncertainty about the exact magnitude of the effect. This underscores the need to interpret the effect size with caution.
